# Views on clinically suspect arthralgia: a focus group study

**DOI:** 10.1007/s10067-015-3038-3

**Published:** 2015-08-15

**Authors:** Elize C. Newsum, Annette H. M. van der Helm-van Mil, Adrian A. Kaptein

**Affiliations:** Department of Rheumatology, Leiden University Medical Centre, C-01-046, PO Box 9600, 2300 RC Leiden, Netherlands; Department of Medical Psychology, Leiden University Medical Centre, Leiden, Netherlands

**Keywords:** Clinically suspect arthralgia, Focus group, Illness perceptions, Patients’ views, Patient-reported outcomes, Quality of life

## Abstract

The rheumatology field is moving towards identifying individuals with an increased risk for rheumatoid arthritis (RA) at a stage when arthritis is still absent but persons having clinically suspect arthralgia (CSA). Incorporating patients’ views in rheumatologic care is pivotal; however, the views of persons with CSA on their condition are unknown. We aimed to help fill this gap by exploring illness perceptions of persons with CSA and their views on hypothetical prognoses for developing RA. Persons with CSA were invited to participate in a semi-structured focus group discussion. Illness perceptions according to the Common Sense Model (CSM) and four a priori formulated themes were explored in detail during the group discussion. The discussion was audio-taped and transcribed verbatim. Transcripts were analysed in an interpretative phenomenological approach manner, on the basis of the dimensions of the CSM by three researchers independently. The views of four participants with CSA were explored during one focus group discussion. Four dimensions of the CSM were mainly observed: Identity, Consequences, Personal Control and Concern. None of the patients identified themselves as being a patient. They did experience pain and impairments in daily functioning and were concerned that their symptoms would progress. In the absence of physician-initiated treatment, some patients changed lifestyle in order to reduce pain and to promote health. Patients unanimously said that they could not interpret prognostic information on RA development expressed in hypothetical chances. Persons with CSA do not consider themselves patients. Prognostic information related to the development of RA based on risk percentages was considered as not useful by persons with CSA. Understanding of the illness perceptions of persons with CSA by health care professionals might improve medical management and facilitate shared decision-making.

## Introduction

Processes underlying rheumatoid arthritis (RA) development are active months to years before arthritis becomes clinically detectable. This has boosted studies on various preclinical disease phases [1]. From a clinical perspective, the phase of symptoms without clinically apparent arthritis is the earliest phase in which RA can be identified [1]. Although factors characterizing RA in this preclinical phase are not yet known, clinical expertise is accurate in identifying individuals with an increased risk for RA [2]. Persons with clinically suspect arthralgia (CSA) are at risk of developing RA according to their rheumatologists [2]. In longitudinal cohorts, individuals with CSA are followed, aiming to identify predictors of RA development [2–5]. These risk factor studies will be followed by intervention trials, evaluating the efficacy of disease-modifying treatment in the preclinical phase of arthralgia.

Labelling individuals as high risk for developing a debilitating illness might elicit feelings of concern, anxiety and depression and consequently reduce individuals’ quality of life. For persons with arthralgia, the first models predicting RA development have been derived [6]. Thus far, the psychological impact of being labelled as susceptible and having a particular risk of developing RA has not been investigated in persons with CSA. Such persons experience their health status and risks differently than health care professionals do, and they have specific thoughts about this. Therefore, knowledge of persons’ views is of importance to improve modern health care where shared decision-making is pivotal. Before launching large-scale quantitative studies on this subject, qualitative research regarding patients’ views (cognitions and emotions) is indicated to explore this issue.

In this light, we aimed to explore in persons with CSA (1) illness perceptions, (2) views regarding four themes about CSA suggested by rheumatologists and (3) views about different (hypothetical) prognoses for developing RA. Given the state of the art regarding these topics, we decided to use a focus group approach to try and answer the three subjects.

## Methods

### Study design

One focus group discussion with a duration of ~2 h was set up at the outpatient clinic of the Rheumatology Department of the LUMC. In an explorative phase, focus group discussions are valuable as discussions between participants allow examining not only what they think but also how they think and why they think that way [7]. In their book “Interpretative Phenomenological Analysis”, Smith, Flowers and Larkin explain how a focus group may allow “participants to express their own personal experiences in sufficient detail and intimacy” [8]. The discussion was chaired by a health psychologist experienced in leading group discussions (AAK). Two investigators (AvdH, rheumatologist; ECN, MD) observed the meeting. The discussion was audio-taped and transcribed verbatim.

The dimensions of the Brief Illness Perception Questionnaire (B-IPQ) and four a priori formulated themes were used as a guideline during the focus group discussion. In addition, the participants were asked to draw their pain due to CSA.

The B-IPQ assesses illness perceptions according to Leventhal’s Common Sense Model (CSM), which postulates that illness perceptions (cognitive and emotional representations of an illness), and coping responses are determinants of disease outcome [9, 10]. Second, asking persons to draw their illness is another approach to explore persons’ views on an illness and its treatment [11–14]. This method has shown that ‘subjective views’ may be a better predictor of medical outcome than ‘objective’ measures [13]. The a priori formulated themes evolved out of discussions with a rheumatologist specialised in research in people with CSA and a medical psychologist. The themes were as follows: perception of having arthralgia and being at risk to develop RA, changed behaviour due to perceived symptoms, concerns about the future and participants’ ideas of different hypothetical prognoses. While we were interested in how a particular risk of developing RA might change participants’ views on their CSA, we proposed several hypothetical prognoses and asked how these influenced their views on CSA. Prognoses proposed were as follows: one out of ten persons with CSA will develop RA, or three out of ten, two out of then persons or two out of three persons will develop RA. We also presented these proportions in percentages.

### Participants

The inclusion criterion for participating in the focus group discussion was being included in the Leiden CSA cohort recently. Within the Leiden Rheumatology outpatient clinic, incident people with CSA are followed longitudinally in a cohort if they have arthralgia of <1 year of hand or foot joints without clinical arthritis at physical examination and an increased risk of developing clinical arthritis according to the rheumatologists [2]. In addition, patients were informed on the suspicion on imminent RA, the unknown actual risk on RA and follow-up [2]. In the CSA cohort disease-modifying anti-rheumatic drug treatment is not prescribed.

Fifty-eight persons with CSA were included in the CSA cohort between August 15 and December 16, 2014, of which 32 randomly selected persons were approached by telephone and asked to participate in the focus group discussion. If interested, a letter with information about the study procedure was sent.

### Data analysis

The focus group transcript was analysed through interpretative phenomenological analysis (IPA) by three researchers independently and coded according to the nine dimensions of the B-IPQ. As the CSM forms the theoretical basis of our study, adhering to IPA is, in our view, perfectly suited in this qualitative study. IPA “is committed to the examination of how people make sense of their major life experiences” and “it is concerned with exploring experience in its own terms” [8]. Differences in coding between the researchers were discussed until consensus was achieved.

The research protocol was approved by the local Medical Ethical Committee of the Leiden University Medical Centre; all participants provided written informed consent.

## Results

### Characteristics of participants

Of the 32 approached persons with CSA, 7 had agreed to participate in the focus group discussion. Unfortunately, three had to cancel due to illness, bronchitis and an ankle distortion on the day the discussion was scheduled. All four participants were female; their age ranged from 24 to 54 years. Both anti-citrullinated peptide antibody (ACPA)-positive and ACPA-negative participants and participants with a positive and negative family history for RA were included. These and other clinical characteristics are summarized in Table 1.

**Table 1:** Characteristics per participant

	1	2	3	4
Age (years)	54	42	30	24
Sex	F	F	F	F
Symptom duration (weeks)	86	50	16	30
68-TJC	10	4	6	9
CRP (mg/L)	<3.0	<3.0	48.5	<3.0
ACPA (anti-CCP2)	+	−	−	−
RF	+	−	−	−
Family history for RA	−	−	−	+

*F* female, *TJC* tender joint count, *CRP* C-reactive protein, *ACPA* anti-citrullinated peptide antibody, *RF* rheumatoid factor, *+* positive, *−* negative

### Illness perceptions

Of the dimensions of illness perceptions, four were frequently observed—Identity, Consequences, Personal Control and Concern—and will be discussed further. Results of the other dimensions of illness perceptions will not be discussed while these were under-represented.

### Identity

Regarding the questions ‘*Do you feel you are ill*?’ and ‘*Are you a patient*?’, all participants unanimously answered ‘*No*’. So, none of them identified themselves as being a patient. Factors that were mentioned if one identified themselves as being a patient were as follows: not being able to do anything, experiencing many limitations in daily life due to symptoms, and necessity of frequent hospital visitations/medical care. One participant described her view as,

*“I feel as fit as a fiddle. The only thing is that now and then my joints opt out. Honestly, I don’t feel like a patient at all”.*

Regarding pain, all participants mentioned experience of pain on a daily or periodical basis.

### Consequences

Participants mentioned different Consequences due to their arthralgia, such as difficulties while putting on one’s shoes and a decrease in the ability to perform hobbies as hiking and making furniture. Social consequences were discussed also, such as avoiding shaking hands due to pain:

*“Shaking hands, that hurts. Particularly when people give a firm handshake”.*

Another participant experienced difficulties in keeping up her social network and her student life because of pain and a lack of understanding about her symptoms in her social environment:

*“However, I do notice that I want to avoid certain situations. For instance, sometimes I put off visitors because I know they won’t understand I am in pain. Or because they don’t take into account that I have to stand up on my feet quite often. Then I prefer to say ‘Well, not today, thank you,’ instead of joining them for an outing”.*

### Personal control

Regarding Personal Control, we observed that the participants had adopted health-promoting behaviour such as dietary changes, haptonomy, yoga and mindfulness. Mindfulness, defined as being attentive to and aware of what is taking place in the present, is a skill that can be learned through practice. The technique is believed to promote well-being [15]. One patient said about mindfulness,

*“Mindfulness really is about reflecting on what’s happening here and now. When you are in pain, you tend to put up a fight against it. And that actually makes things worse and the pain becomes a big thing. You could also take a different attitude and say, let’s embrace the pain. And then the pain isn’t so bad after all”.*

### Concern

Overall, participants mentioned concerns about pain, uncertainty of pain progression, developing functional limitations and prognosis. One participant feared her unpredictable pain attacks and worried about the uncertainty of pain progression:

*“These are attacks of pain that come and go. And talking about fear, you continually think, when will the pain be back, when will it return? The pain is really killing”.*

*“But when I suffer this much now, what’s going to happen next? What kind of pain is still in store for me? What will this lead to? I’m inclined to think; now I’m playing Russian roulette, but eventually it will be outright war”.*

A participant with a positive family history for RA developed a detailed fear about RA symptoms and related limitations:

*“My aunts have red hands that are swollen. They are unable to do anything. And that is my nightmare, that I won’t be able to do anything anymore. Oh my God, I have this bad dream of me having these big swollen hands. It really is a spectre, of these claws, my hands that I am unable to open any longer”.*

Another participant took a more positive view. In her belief, her arthralgia would not develop into RA:

*“Maybe it will pass, but it may also develop into rheumatism. So I still have this hope that in the future it may just pass away. So let’s go for it”.*

### Drawings

**Fig. 1:**
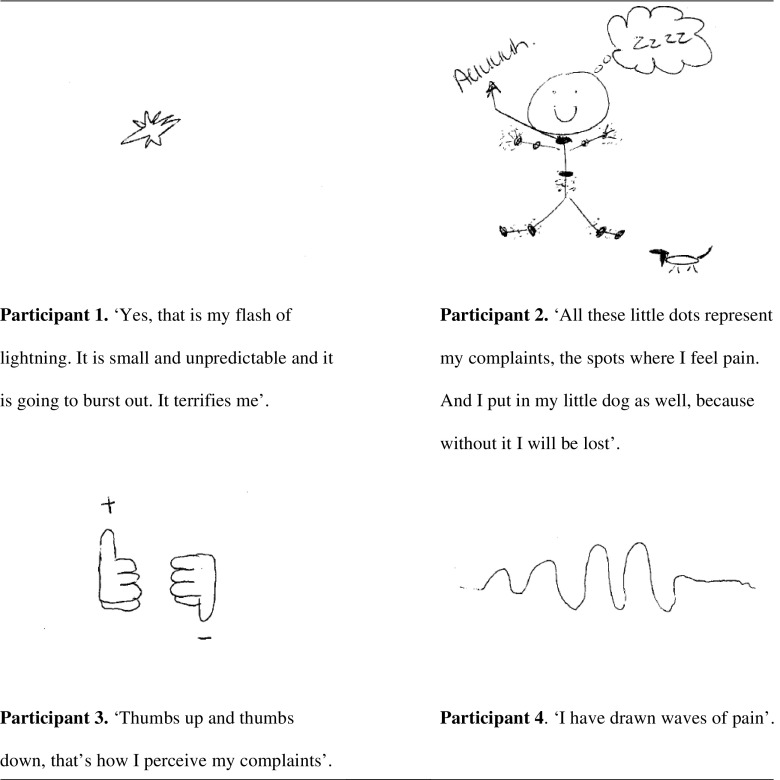
Participants’ drawings of their pain due to arthralgia

We asked the participant to draw their pain. In these drawings, several dimensions of the illness perceptions can be recognized. Three participants used a symbolic way of presenting their arthralgia, namely, a flash of lightning, thumbs up and down, and waves of pain. The flash of lightning expresses the uncertainty of the unpredictable pain attacks one participant experienced, which can be interpreted as reflections of the dimensions Identity, Concern and Timeline. Identity and Timeline are also represented in the drawing of waves of pain, reflecting the fluctuating intensity of pain over time. Another drawing also reflects the dimension Identity. The participant drew herself and her tender joints in detail, combined with written expressions of pain and fatigue. Figure 1 represents the drawings combined with participants given explanation.

### Participants’ views on prognoses

Regarding the participants’ ideas on different (hypothetical) prognoses to develop RA, we observed that they preferred to have information on the origin of their symptoms. In addition, all participants unanimously said that they could not interpret prognostic information as expressed in proportions or in chances that the CSA would progress to RA. One participant said,

*“Statistics like 1 out of 10 really don’t mean a thing to me. The way I reason is, I am not 1 out of 10. That’s how I feel about it, it won’t be me”. “Percentages, they don’t mean a great deal to me”.*

## Discussion

In this study, we explored illness perceptions of persons suffering from CSA about their arthralgia. We also examined the impact of their symptoms on daily life; furthermore, their thoughts about chances to develop RA were explored. The so-called Common Sense Model was applied as this is a solid theoretical model with strong empirical support. The major results of our theory-driven study are that persons with CSA perceive their condition to be dominated by pain and impairments in daily functioning. As a consequence, some of them changed their lifestyle in order to reduce pain and to promote health. Most participants were concerned about the progression of their symptoms, but none of the participants did identify themselves as ‘patients’, as being a patient was perceived as a condition determined by having more functional limitations and the necessity of regular medical care.

Interestingly, previous research on illness perceptions in patients with RA revealed that the dimensions Consequences, Identity and Concern were the most significant correlates of their physical health-related quality of life [16]. Our study shows that these dimensions also do play an important role already in the preclinical phase of arthralgia. Previous research in patients with RA comparing negative and positive representations of disease revealed that the group characterised by a negative representation of their illness attributed more symptoms to their condition, reported stronger perceptions of the dimensions Consequences and Chronicity and reported lower Control compared to the positive representation group[17]. Therefore, negative illness perceptions of CSA patients might be important targets in research that aims at improving quality of life in an earlier stage of the disease.

There is an increasing interest in identifying individuals with arthralgia at risk for RA [2, 5, 6]. In modern medicine, there is a tendency to diagnose and treat earlier with the purpose to stop the development of a debilitating disease. Although diagnosing RA in a phase of CSA is not possible yet, physicians, including rheumatologists, generally adhere to explore risk factors and to apply prognostic models. For persons with arthralgia, the first models predicting RA development have been derived [6]. We observed that persons with CSA perceived prognostic information related to the development of RA based on risk percentages as not useful. They felt that such information did not guide their perceptions or potential treatment decisions as it does not include a ‘yes or no’ answer. These results imply that prognostic models are not optimally suited for shared decision-making and that a diagnostic strategy, allowing diagnosing RA in a symptomatic preclinical phase, is preferred by these persons with CSA.

This study has some limitations. The major limitation is that our final sample size was small. However, this study is the first providing insight in the illness perceptions of persons with arthralgia who are at risk for progression to RA. Despite the small number of participants, the consensus of opinion about discussed dimensions was evident, and the unanimity of the participants’ views on the main findings strengthens the validity of our findings. Notably, we included both ACPA-positive and ACPA-negative participants and participants with a positive and negative family history for RA. The clinical and medical characteristics of the studied participants are in line to those of the larger CSA cohort [2], suggesting against selection bias. Larger, quantitative studies are now required. The transcripts were coded by three researchers independently to ensure reliability of coding. Hence, our analysis seems to be a reliable way of avoiding personal interpretation of the researcher.

In conclusion, our study offers useful insight in the views of persons with CSA about illness perceptions regarding diagnosis and behavioural changes. In our view, the main contribution of our study lies in better understanding of how persons at risk for developing a debilitating disease make sense of physical symptoms. The observation that persons with CSA do not consider themselves as patients suggests that the fact that they are being followed up by rheumatologists on their disease course was not harmful with respect to their perception of their symptoms or identity. If rheumatologists in their communication with persons with CSA explore their illness perceptions, negative illness representations might be identified. This may allow addressing and changing the illness perceptions in a more constructive direction. This in turn may be a first step in improving quality of life. Furthermore, understanding of the illness perceptions of people with CSA by health care professionals might improve medical management and facilitate shared decision-making.
